# Necrostatin-1 alleviates temporomandibular joint osteoarthritis and inhibits chondrocyte senescence and necroptosis possibly via p53/ MAPK downregulation

**DOI:** 10.3389/fbioe.2025.1735484

**Published:** 2025-12-18

**Authors:** Ying Zhang, Wenyi Cai, Antong Wu, Rui Li, Xin Li, Kaihan Zheng, Yufu Lin, Qingbin Zhang, Wei Cao

**Affiliations:** 1 Department of Temporomandibular Joint, School and Hospital of Stomatology, Guangdong Engineering Research Center of Oral Restoration and Reconstruction, Guangzhou Key Laboratory of Basic and Applied Research of Oral Regenerative Medicine, Guangzhou Medical University, Guangzhou, China; 2 Department of Stomatology, The Second Affiliated Hospital, Guangzhou Medical University, Guangzhou, China; 3 Laboratory for Myology, Department of Human Movement Sciences, Faculty of Behavioural and Movement Sciences, Vrije Universiteit Amsterdam, Amsterdam Movement Science, Amsterdam, Netherlands

**Keywords:** temporomandibular joint osteoarthritis, necrostatin-1, cellular senescence, necrosis, p53, MAPK

## Abstract

Temporomandibular joint osteoarthritis (TMJ-OA) is a prevalent oral and maxillofacial disorder characterized by a complex etiology and pathogenesis. Targeting and eliminating senescent chondrocytes is emerging as a promising therapeutic strategy for TMJ-OA. Necrostatin-1 (NEC1), a receptor-interacting protein kinase 1 (RIPK1)-targeted necroptosis inhibitor, has shown potential therapeutic effects in various skeletal disorders. However, the therapeutic implications of NEC1 in TMJ-OA, particularly regarding its effect on chondrocyte senescence, remain unclear. In this study, models of TMJ-OA were established by utilizing monosodium iodoacetate (MIA) to induce TMJ-OA in rats. NEC1 was administered via intra-articular injection. Treatment with NEC1 significantly alleviated the progression of TMJ-OA in rats, reduced pain, and restored cartilage integrity. *In vitro*, NEC1 (40 μM) exhibited cytoprotective effects on Rat primary condylar chondrocytes (rPCCs), preserving cell viability and reducing cellular senescence markers (Cdkn1a and Cdkn2a) following IL-1β+TNF-α treatment. Additionally, NEC1 inhibited the expression of extracellular matrix (ECM) degradation markers (MMP3, MMP9, MMP13) and programmed necrosis indicators (p-MLKL) in chondrocytes. NEC1 downregulated inflammation-induced p53 and MAPK signaling pathways in rPCCs.Thus, NEC1 demonstrated a significant capacity to slow the progression of TMJ-OA by inhibiting and alleviating condylar chondrocyte senescence, necrosis, and ECM degradation, suggesting its potential role in the treatment of TMJ-OA.

## 1 Introduction

Temporomandibular joint osteoarthritis (TMJ-OA) is a chronic degenerative disease affecting the temporomandibular joint (TMJ), with high prevalence among both adolescents and the elderly. The primary clinical symptoms include TMJ pain, limited mouth opening, and joint noise. TMJ-OA causes severe joint damage, potentially leading to joint ankylosis, facial deformities, or occlusal disorders (Cardoneanu et al., 2022). The major treatment of TMJ-OA currently aims to relieve TMJ symptoms, slow TMJ-OA progression, and improve TMJ function, although these methods are mainly effective in the early stages (Delpachitra and Dimitroulis, 2022). Treatment strategy, e.g., rehabilitation, medicine, TMJ injection, occlusal splint, and surgery, in which are still limited to treat TMJ-OA (Wang et al., 2015).

The pathogenesis of TMJ-OA is complex, with several contributing factors, among which aging plays a critical role. Studies have highlighted multiple mechanisms linking TMJ-OA to joint aging, including age-related inflammation also known as ‘inflammaging’, cellular senescence characterized by the senescence-associated secretory phenotype (SASP), mitochondrial dysfunction, and oxidative stress (Ye et al., 2024). Aging contributes to biological changes, such as cell cycle arrest, that induce chronic inflammation and extracellular matrix (ECM) degradation (Xu et al., 2024). SASP is associated with necrosis, and senolytics treatment also inhibits cell necrosis and inflammation (Thadathil et al., 2022). Therapeutic agents inhibiting cellular senescence and necroptosis for TMJ-OA treatment have not been reported yet.

Necrostatin-1 (NEC1) is a receptor-interacting protein kinase-1 (RIPK1)-targeted necroptosis inhibitor that has shown potential therapeutic effects in various skeletal system diseases, including cartilage thinning, osteonecrosis, osteoporosis, and OA (L. Cao and Mu, 2021). Meanwhile, NEC1 also exhibits beneficial effects on a range of aging-related diseases, such as brain ischemic necrosis, acute myocardial infarction, Alzheimer’s disease, neuro-inflammation, cognitive dysfunction, and liver ischemia-reperfusion injury (Deng et al., 2019; Garvin et al., 2018; Qing et al., 2018; Yang et al., 2019; Zhang et al., 2023; Zhong et al., 2020a). Aging-related SASP induces cellular necrosis and cellular necrosis is also related to aging and inflammation (Liu et al., 2024). However, the effect of NEC1 treatment on TMJ-OA and underlying mechanisms remains unclear.

Hence, this study aimed to investigate whether NEC1 can alleviate the inflammatory response, cartilage ECM degradation, cellular senescence, and necrosis in TMJ-OA. Monosodium iodoacetate (MIA)-induced rat TMJ-OA was developed and NEC1 was injected into joint cavity to treat TMJ-OA. The effects of NEC1 on rat primary condylar chondrocytes (rPCCs) *in vitro* was investigated. Furthermore, it sought to explore the molecular regulatory mechanism of NEC1 on the p53/MAPK signaling pathways, in order to propose new directions for clinical treatment. Our findings shown that NEC1 can alleviate TMJ-OA and inhibit chondrocyte senescence and necroptosis possibly *via* p53/MAPK downregulation.

## 2 Materials and methods

### 2.1 Animal study

Sprague–Dawley (SD) rats were purchased from Guangdong Medical Laboratory Animal Center. A total of 15 SD rats (8 weeks old, female) with an average weight of 200 g were used in this study. The rats were randomly divided into three groups (n = 5): control group, MIA + NS group, and MIA + NEC1 group. The MIA-induced TMJ-OA rat model was established as previously described (Wang et al., 2012). In brief, 0.5 mg MIA (Sigma, USA) was dissolved in 50 μL normal saline (NS) and injected with an insulin needle into the bilateral TMJ. After 2 weeks of TMJ-OA induction, the rats received intra-articular injections once a week for 4 weeks. The MIA + NS group was injected with 50 μL NS, and the MIA + NEC1 group was injected with 50 μL NEC1 (40 μM; MedChemExpress, USA). After completion of the experiment, all rats were euthanized *via* rapid intraperitoneal injection of pentobarbital sodium, following established protocols (Sukarawan et al., 2023). Briefly, pentobarbital sodium was administered at a dose of 120 mg/kg, based on animal weight. The solution was injected into the lower right abdomen using a sterile syringe to ensure accurate delivery. Following the injection, animals were monitored for the cessation of vital signs, including spontaneous breathing, heartbeat, and corneal reflexes. To ensure death, cervical dislocation was performed as an additional measure. This sacrifice method ensured a humane and painless euthanasia process, in compliance with ethical standards of animal welfare. All experimental procedures described in this study were approved by the Experimental Animal Ethics Committee of Guangdong HUA WEI Testing Co., Ltd (No.202209011).

### 2.2 Micro-CT analysis and behavioral tests in rats

The TMJ tissue samples were harvested and fixed with 4% paraformaldehyde (PFA) for 48 h, then replaced with 70% ethyl alcohol. The samples were scanned coronally at 90 kV and 65 μA with a 20 μm-effective pixel size using Micro-CT (VENUS, China). The ratio of bone volume to total tissue volume (BV/TV), trabecular separation (Tb.Sp), and trabecular thickness (Tb.Th) were analyzed to investigate bone structure parameters.

Head withdrawal threshold (HWT) was measured once a week. Mechanical hyper-nociception in the TMJ region was assessed using Von Frey filaments (IITC, USA). After acclimatization, a series of increasing-force filaments were applied to the preauricular area of each rat. A positive response was recorded if the rat exhibited a head retraction or swinging motion. HWT was defined as the minimal force that elicited a withdrawal response in at least three consecutive trials. HWT measurements were taken once per week.

### 2.3 Histology

After the Micro-CT scanning, the TMJ tissue samples were decalcified at room temperature in 10% Ethylene Diamine Tetraacetic Acid (EDTA) for 6 weeks. The samples were then dehydrated, embedded in paraffin, and sectioned at a thickness of 5 μm. The sections were stained with hematoxylin and eosin (H&E, Solarbio, China) and safranin-O and fast green (S&F, Solarbio, China). The degree of articular cartilage degeneration was assessed using the Mankin score system (Takahashi et al., 2019). Images were captured with a light microscope (Leica, Germany).

### 2.4 Immunohistochemistry

Immunohistochemistry staining was performed using anti-MMP13 (1:100, Thermo Fisher, USA) and anti-COL2A1 (1:500, Arigobio, China). In brief, the paraffin sections were dewaxed and rehydrated, then incubated overnight at 4 °C with the primary antibody. The sections were washed 3 times with PBS and incubated with the secondary antibody for 1 h at room temperature. Finally, DAB (diaminobenzidine, Beyotime, China) solution was added, and the color development time was carefully monitored under the microscope. Positive staining was indicated by a brownish-yellow color. After counterstaining with haematoxylin, the sections were dehydrated and sealed with neutral resin. Images were captured with a light microscope (Leica, Germany). The specific methods and materials were based on our previous study (Wu et al., 2023).

### 2.5 Rat primary condylar chondrocytes (rPCCs) culture and treatment

According to the standard protocol reported (Zhang et al., 2019), rPCCs were isolated from 3-week-old SD female rats (Guangdong Medical Laboratory Animal Center), and third passage cells were used for follow-up experiments. Briefly, condylar tissue blocks were sectioned into 1 mm^3^ pieces and digested with 0.2% collagenase II (Sigma-Aldrich, USA) for 4 h at 37 °C. Subsequently, rPCCs were obtained after centrifugation, resuspended, and cultured (culture medium: DMEM/F12 + 10% FBS +1% Penicillin-Streptomycin, Gibco, USA). rPCCs were identified by toluidine blue staining (Solarbio, China) and immunofluorescence (IF, anti-COL2A1, 1:100, Abcam, UK). Primary chondrocytes were isolated from rat condylar cartilage tissues and confirmed by toluidine blue staining and immunofluorescence. The cells were positive for Col2a1 and toluidine blue, indicating their identity as chondrocytes. To assess the cytoprotective effects of NEC1 on chondrocytes and to determine the optimal *in vitro* concentration, rPCCs were treated with various concentrations of NEC1 (20, 40, 80, and 160 μM) for 24, 48, and 72 h, followed by the CCK8 assay.

*In vitro* experiments were divided into three groups: Control group, TMJ-OA group, and TMJ-OA + NEC1 (40 μM) group. The TMJ-OA model was established by treating the rPCCs with 10 ng/mL IL-1β plus 20 ng/mL TNF-α (Sino Biologicals, China) for 48 h. The control group was cultured in a normal culture medium, and the TMJ-OA + NEC1 group was pretreated with NEC1 (40 μM) for 1 h before induction with inflammatory factors.

### 2.6 Cell viability assay

The Cell Counting Kit-8 (CCK8; Dojindo, Japan) was employed to assess the cytotoxicity and proliferation effects of various treatments on rPCCs. In brief, rPCCs were plated in a 96-well plate at a density of 5,000 cells per well and exposed to NEC1 at concentrations of 20, 40, 80, and 160 μM, or to a combination of 10 ng/mL IL-1β and 20 ng/mL TNF-α for durations of 24 and 48 h, respectively. Subsequently, 10 μL of the CCK8 working solution was added to each well, and after a 2-h incubation period, the absorbance values (optical density, O.D.) at 450 nm were recorded using a microplate reader (Thermo Fisher, USA).

### 2.7 Immunofluorescence

rPCCs were plated in 24-well plates at a density of 25,000 cells per well. Following treatment according to the specified groups, the cells were first fixed with 4% paraformaldehyde (PFA) for 15 min, then permeabilized with 0.5% Triton X-100 for 5 min, and blocked with 3% bovine serum albumin (BSA) for 30 min at room temperature. Subsequently, the cells were incubated overnight at 4 °C with anti-MMP3 (1:100; Affinity, China) or anti-MMP13 (1:100; Affinity, China). On the following day, the cells were further incubated at room temperature for 1 h with FITC-conjugated secondary antibodies (1:200; Proteintech, China), then stained with DAPI, and imaged using a fluorescence microscope (Nikon, Japan). For IHC analysis, regions of interest (ROI) were selected within the condylar cartilage to ensure representative the functional zones. Quantification was performed using ImageJ.

### 2.8 Senescence-associated β-galactosidase (SA-β-Gal) staining

Cells were stained using the SA-β-Gal staining kit (Beyotime, China) according to the manufacturer’s instructions. In brief, the treated rPCCs were fixed with 4% paraformaldehyde (PFA) for 15 min, then incubated with the staining solution in a CO_2_-free incubator at 37 °C for 12 h. Senescent cells were visualized as blue. Images were acquired using an optical microscope (Leica, Germany).

### 2.9 Western blot

To prepare protein samples, cells were lysed using RIPA lysis buffer (Beyotime, China) containing 1% protease and phosphatase inhibitors (Beyotime, China) for 30 min. The lysate was then collected and centrifuged at 14,000 rpm for 15 min at 4 °C to obtain the total protein. The concentration of total protein was measured using a BCA protein assay kit (Beyotime, China). For Western blotting, protein samples were first separated by SDS-PAGE and transferred onto polyvinylidene difluoride (PVDF) membranes (Millipore, USA). The PVDF membranes were then blocked with QuickBlock™ Blocking Buffer (Beyotime, China) and incubated overnight with a primary antibody at 4 °C. Following this, the membranes were incubated with a secondary antibody for 1 h at room temperature. Finally, protein bands were visualized using an Ultra-sensitive ECL ChemiLuminescence kit (Beyotime, China). The brands and concentrations of the primary antibodies used in the WB experiment are shown in Table 1.

**TABLE 1:** Primary antibodies.

Antibody	Dilution	Manufacturer
anti-MMP9	1:1000	Abcam, UK
anti-p-MLKL	1:1000	Thermo Fisher, USA
anti-p38 MAPK	1:1000	CST, USA
anti-p-p38 MAPK	1:1000	CST, USA
anti-ERK1/2	1:1000	CST, USA
anti-p-ERK1/2	1:1000	CST, USA
anti-p53	1:1000	CST, USA
anti-p21	1:1000	Proteintech, China

### 2.10 Real-time quantitative polymerase chain reaction (RT-qPCR)

Total RNA was extracted from cells using the RNA Extraction Kit (EZBioscience, China). cDNA was synthesized with 500 ng of total RNA at 37 °C for 15 min using 5×EvoM-MLV RT Master Mix (AG, Guangzhou, China). RT-qPCR was then conducted on an RT-PCR machine (Agilent Technologies, USA) with SYBR Green Master Mix (EZBioscience, China). The sequences of the PCR primers are listed in Table 2.

**TABLE 2:** Primers sequences.

Gene	Forward primer (5′-3′)	Reverse primer (5′-3′)
*Gapdh*	GCCTTCCGTGTTCCTACC	CCT​GCT​TCA​CCA​CCT​TCT​T
*Mmp3*	TGAAGATGACAGGGAAGC	CTGGAGAATGTGAGTGGG
*Mmp13*	TGACCCAGCCCTATCCCT	ACC​CTC​CAT​AAT​GTC​ATA​CCC
*Mmp9*	AAG​GAT​GGT​CTA​CTG​GCA​CA	TTG​CGT​TTC​CAA​AGT​AAG​TG
*Cdkn1a*	TTG​CCA​CTT​CTT​ACC​TGG​GG	GTG​ACA​AGG​AGA​CCC​CGA​G
*Cdkn2a*	GAT​GGG​CAA​CGT​CAA​AGT​GG	GAT​GGG​CAA​CGT​CAA​AGT​GG

### 2.11 RNA sequencing

The total RNA of condylar samples was extracted using the Trizol Plus RNA Purification Kit (Thermo Fisher, USA). RNA concentration and purity were measured with a Nanodrop 2000 instrument (Thermo Fisher, USA). After RNA quality control, Shenzhen Haiyi Time Gene Technology Co., Ltd. completed the library construction, RNA sequencing, and data analysis. The specific methods and materials were based on our previous study (Zhong et al., 2020b).

### 2.12 Statistical analysis

All data are expressed as mean ± standard deviation (SD). Statistical analysis of the experimental data was performed using one-way ANOVA and t-tests. Statistical significance was defined as *p* ≤ 0.05. Data visualization was conducted using GraphPad Prism 9 (GraphPad Software, USA).

## 3 Results

### 3.1 NEC1 alleviated the progression of TMJ-OA *in vivo*

**FIGURE 1:**
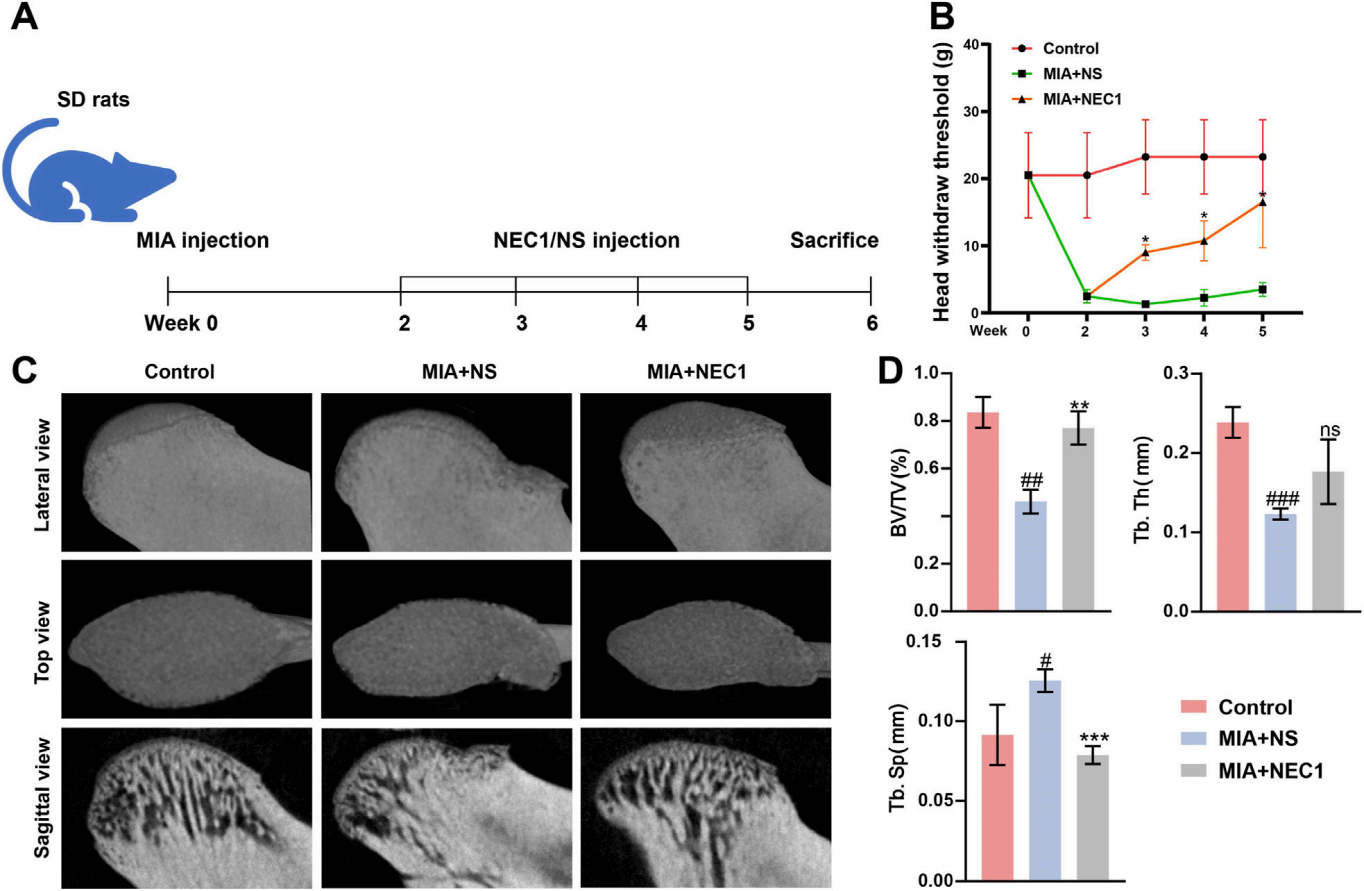
Necrostatin-1 (NEC1) enhanced bone remodeling and reduced pain responses in rats with temporomandibular joint osteoarthritis (TMJ-OA) induced by monosodium iodoacetate (MIA). **(A)** Experimental timeline for TMJ-OA induction and NEC1 treatment *in vivo*. **(B)** Time-dependent pain behavior in rats during the experiment before MIA induction and treatment with NEC1 from 0 to 5 weeks. **(C)** Micro-CT images of condylar morphology. **(D)** Quantitative analysis of subchondral bone parameters, including Tb.Th, BV/TV, and Tb.Sp. Data were analyzed by one-way ANOVA, n ≥ 3. ^#^ *p* < 0.05, ^##^ *p* < 0.01, ^###^ *p* < 0.001 compared to the control group; *p < 0.05, **p < 0.01, ***p < 0.001 compared to the MIA + NS (normal saline) group. ns indicates no significance.

To assess the therapeutic efficacy of NEC1 in TMJ-OA *in vivo*, a rat with TMJ-OA model was established. Schematic outlines *in vivo* procedure for TMJ-OA induction and NEC1 treatment (Figure 1A). Behavioral tests indicated that rats with TMJ-OA induced by MIA exhibited pronounced sensitivity to mechanical stimulation; However, pain responses in the TMJ area were significantly reduced in the NEC1-treated group compared to the NS group (Figure 1B). Micro-CT imaging (Figure 1C) revealed significant cartilage and subchondral bone damage due to MIA injection. However, NEC1 treatment effectively counteracted these changes. Quantitative analysis of subchondral bone parameters from micro-CT data showed a decrease in trabecular thickness (Tb.Th) and bone volume-to-total volume ratio (BV/TV), along with an increase in trabecular separation (Tb.Sp) in the MIA + NS group compared to the control (Figure 1D). NEC1 treatment significantly ameliorated these bone alterations induced by MIA TMJ-OA modeling.

**FIGURE 2:**
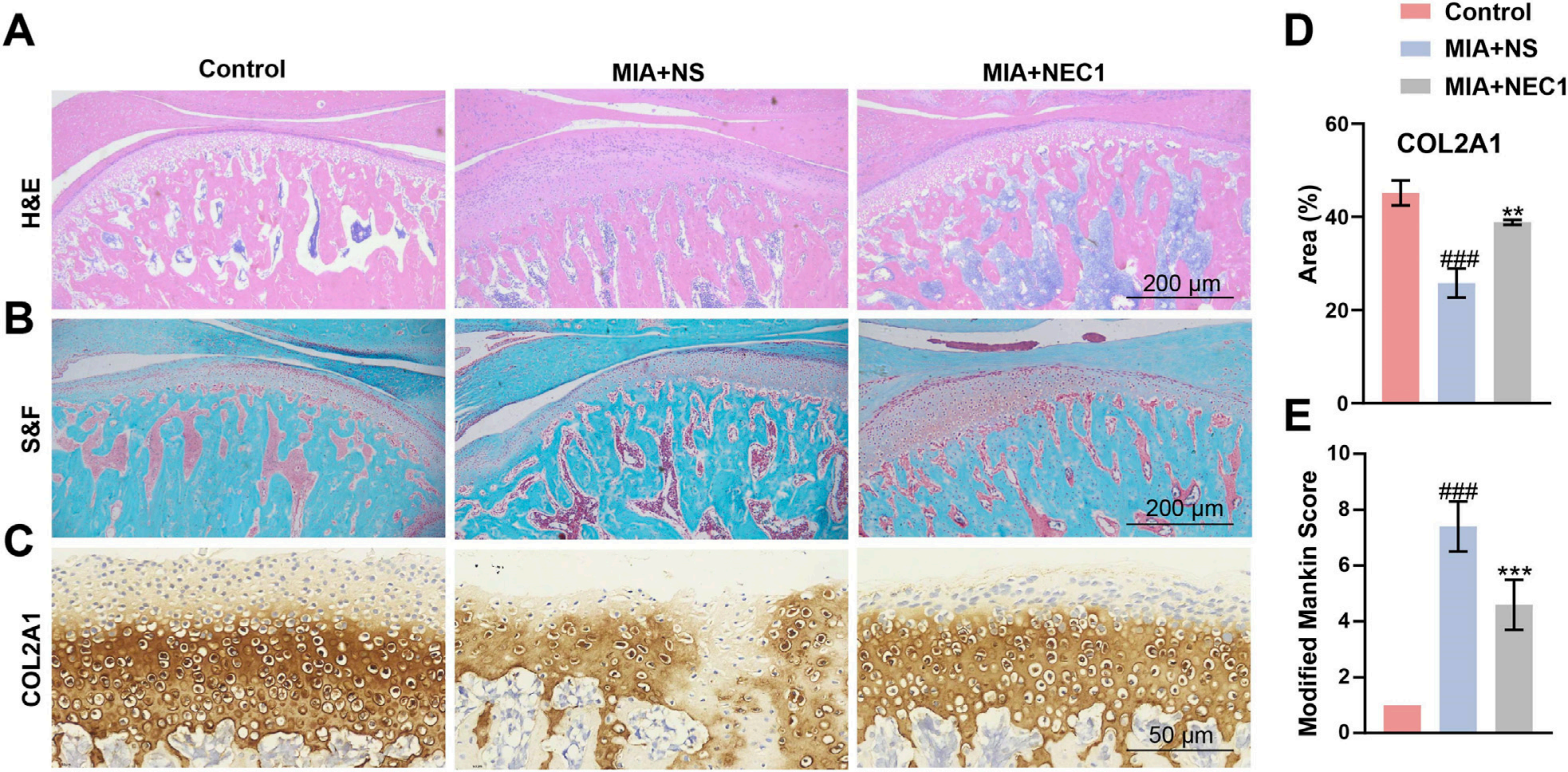
Necrostatin-1 (NEC1) enhanced condylar cartilage reconstruction and COL2A1 expression. **(A)** Representative images of H&E.-stained TMJ of rats with or without NEC1 for 4 weeks **(B)** S&F-stained sections of the joint. **(C)** Immunohistochemistry stained COL2A1 area (brown). **(D)** Quantification of COL2A1 positive area in condylar untreated or treated with NEC1 for 4 weeks **(E)** Modified Mankin scores. Data were analyzed by one-way ANOVA, n ≥ 3. ^###^ *p* < 0.001 compared to the control group; **p < 0.01, ***p < 0.001 compared to the MIA (Monosodium iodoacetate)+NS (Normal saline) group.

Histological analysis using H&E and S&F staining revealed characteristic cartilage and subchondral bone destruction in the MIA + NS group. Conversely, the NEC1-treated group showed notable restoration of TMJ anatomical structure. Articular and calcified cartilage remained relatively intact, subchondral bone degeneration was mitigated, chondrocyte density increased, and proteoglycan staining in the condylar cartilage layer was enhanced (Figures 2A,B). Immunohistochemistry analysis demonstrated that Collagen II (COL2A1), a fibrillar collagen present in cartilage, was reduced in the condylar cartilage of the MIA + NS group compared to the control group. In contrast, COL2A1 was restored in the TMJ following NEC1 treatment (Figures 2C,D). The Modified Mankin scores of each group are illustrated in (Figure 2E). We observed a significant increase in the Mankin score in the TMJ-OA group following MIA treatment compared to the control group. In contrast, the Mankin score in the NEC1 treatment group was significantly decreased compared to the MIA + NS treatment group.

### 3.2 Identification of condylar chondrocytes and effect of NEC1 on cell viability

**FIGURE 3:**
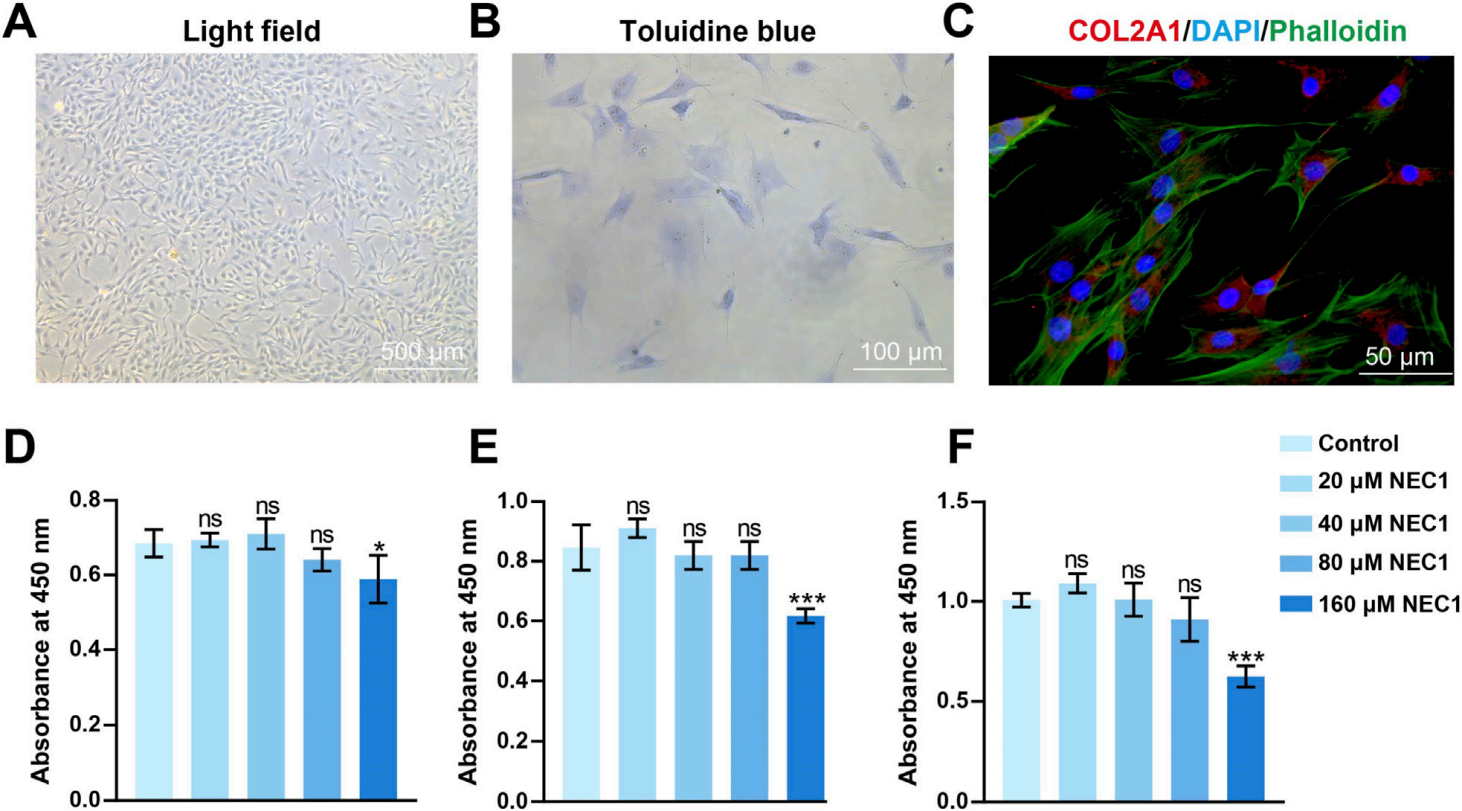
Identification of condylar chondrocytes and cytotoxic effects of Necrostatin-1 (NEC1) on chondrocyte viability. **(A)** Rat primary condylar chondrocytes (rPCCs) were viewed using a light microscope. **(B)** Chondrocytes stained with toluidine blue (blue). **(C)** DAPI-stained nuclei (blue), phalloidin-stained F-actin (green), and COL2A1-strained (red) in chondrocytes with immunofluorescence staining. **(D–F)** rPCCs were treated with NEC1 (20, 40, 80, and 160 μM) for 24, 48, and 72 h, and viability was evaluated by CCK-8 assay. Data were analyzed by one-way ANOVA, n = 3. *p < 0.05, ***p < 0.001 compared to the control group; ns indicates no significance.

rPCCs was collected from rats TMJ. Chondrocytes stained with toluidine blue and COL2A1-strained red, which indicated rPCCs identification (Figures 3A–C). The results demonstrated that NEC1 concentrations up to 80 μM did not exhibit significant cytotoxicity to chondrocytes at any of the time points tested. However, increasing the concentration of NEC1 to 160 μM significantly reduced cell viability, with statistical significance observed (Figures 3D–F). Accordingly, based on these findings and previous research on NEC1 (Cao et al., 2023), a concentration of 40 μM was selected for use in subsequent experiments.

### 3.3 NEC1 alleviated inflammation-induced chondrocyte senescence *in vitro*

**FIGURE 4:**
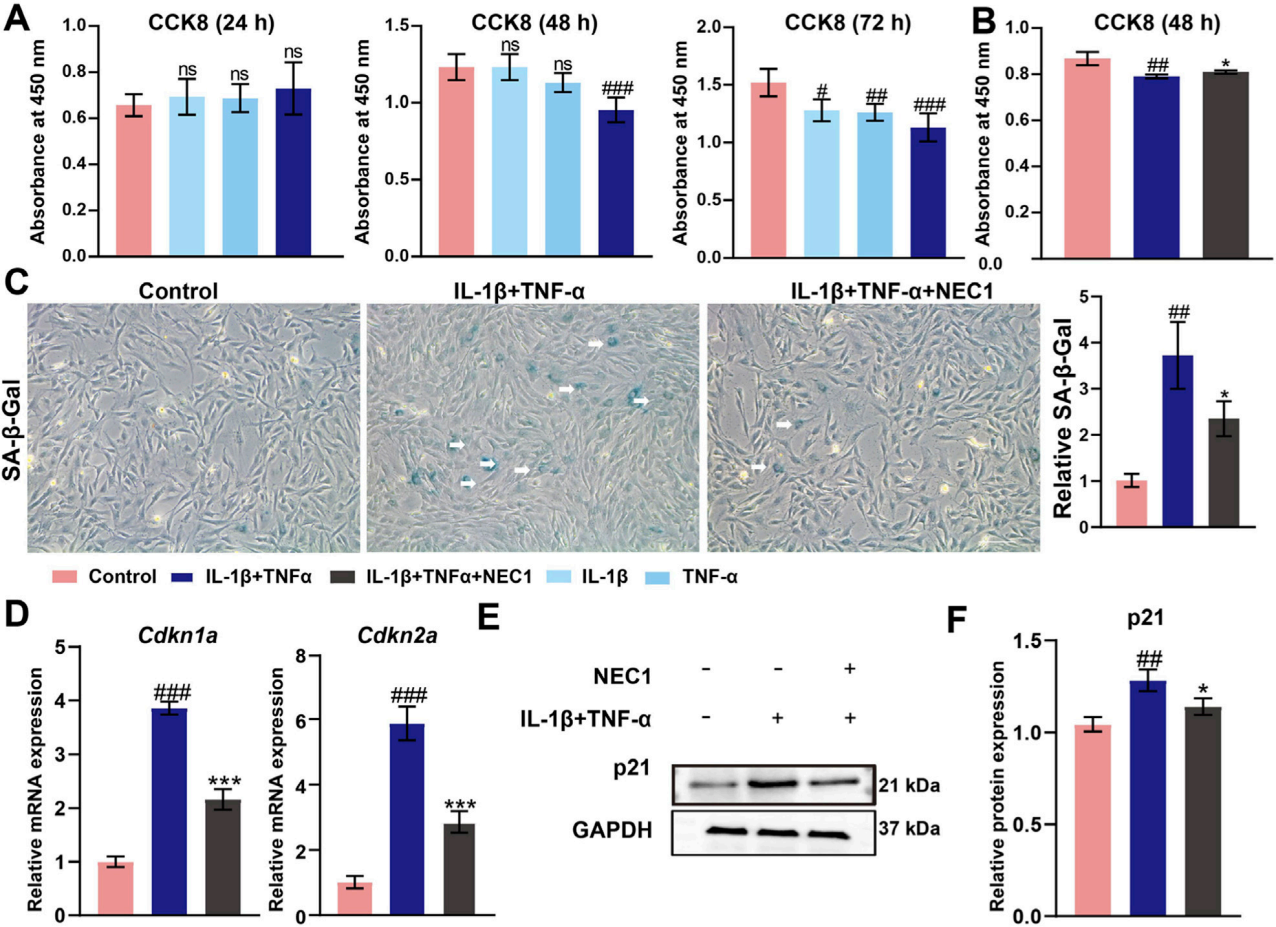
Necrostatin-1 (NEC1) reduced cellular senescence in chondrocytes. **(A)** and **(B)** Cell viability in rPCCs (Rat primary condylar chondrocytes) were evaluated by CCK-8 assay. **(C)** Cell senescence assessed by SA-β-Gal staining (blue) and quantification. **(D)** The expression of cell senescence markers *Cdkn1a* and *Cdkn2a*. **(E)** The expression of p21. **(F)** Quantification of p21. Data were analyzed by one-way ANOVA, n ≥ 3. ^#^ *p* < 0.05, ^##^ *p* < 0.01, ^###^ *p* < 0.001 compared to the control group; *p < 0.05, ***p < 0.001 compared to the IL-1β and TNF-α group. ns indicates no significance.

Cytokines such as IL-1β, TNF-α, or their combination are commonly employed to establish *in vitro* models of TMJ-OA (Chen et al., 2024; DiNicola et al., 2024; Yang et al., 2024) (Figures 4A,B). In TMJ-OA, chondrocytes exhibit a senescent phenotype, characterized by elevated expression of SASPs, DNA damage, and cell cycle arrest. SA-β-Gal staining revealed that NEC1 treatment significantly reduced the frequency of senescent chondrocytes following IL-1β and TNF-α stimulation (Figure 4C). IL-1β and TNF-α significantly upregulate the expression of cell senescence markers p21 (*Cdkn1a)* and p16 (*Cdkn2a)* (Figures 4D–F).

### 3.4 NEC1 reduced necrosis and extra cellular matrix (ECM) catabolism *in vitro*

**FIGURE 5:**
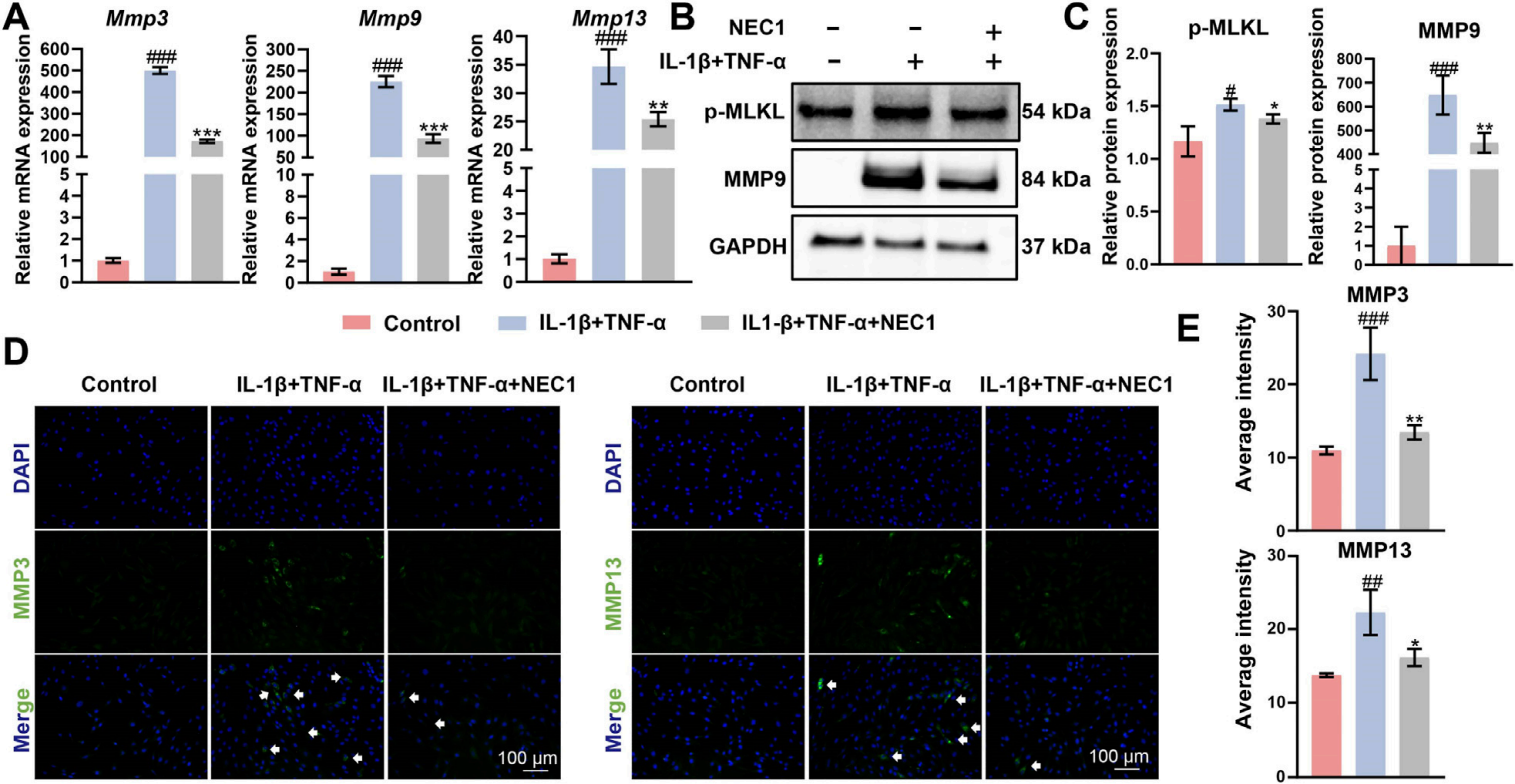
Necrostatin-1 (NEC1) reduced necrosis and extra cellular matrix catabolism in chondrocytes. **(A)** mRNA levels of *Mmp3*, *Mmp9*, and *Mmp13* detected by RT-qPCR. **(B,C)** Protein levels of p-MLKL and MMP9 detected by Western blot. **(D,E)** Protein levels of MMP3 and MMP13 detected by immunofluorescence. Data were analyzed by one-way ANOVA, n ≥ 3. ^#^ *p* < 0.05, ^##^ *p* < 0.01, ^###^ *p* < 0.001 compared to the control group; *p < 0.05, **p < 0.01, ***p < 0.001 compared to the IL-1β and TNF-α group. ns indicates no significance.

These inflammatory stimuli significantly upregulated the expression of ECM degradation markers, specifically MMP3, MMP9, and MMP13. NEC1 treatment effectively reduced the expression of these markers at both the RNA and protein levels, as confirmed through qPCR, Western blot, and immunofluorescence analyses (Figure 5). These results indicate that NEC1 may help mitigate ECM degradation in inflammatory environments, suggesting a potential role in preserving cartilage integrity. Additionally, p-MLKL serves as a crucial marker of programmed necrosis, in which MLKL undergoes activation and phosphorylation (forming p-MLKL) before translocating to the cell membrane. This translocation results in membrane rupture and cell death, making p-MLKL a key indicator for confirming programmed necrosis.

### 3.5 NEC1 inhibited P53/MAPK signaling pathway *in vitro*

**FIGURE 6:**
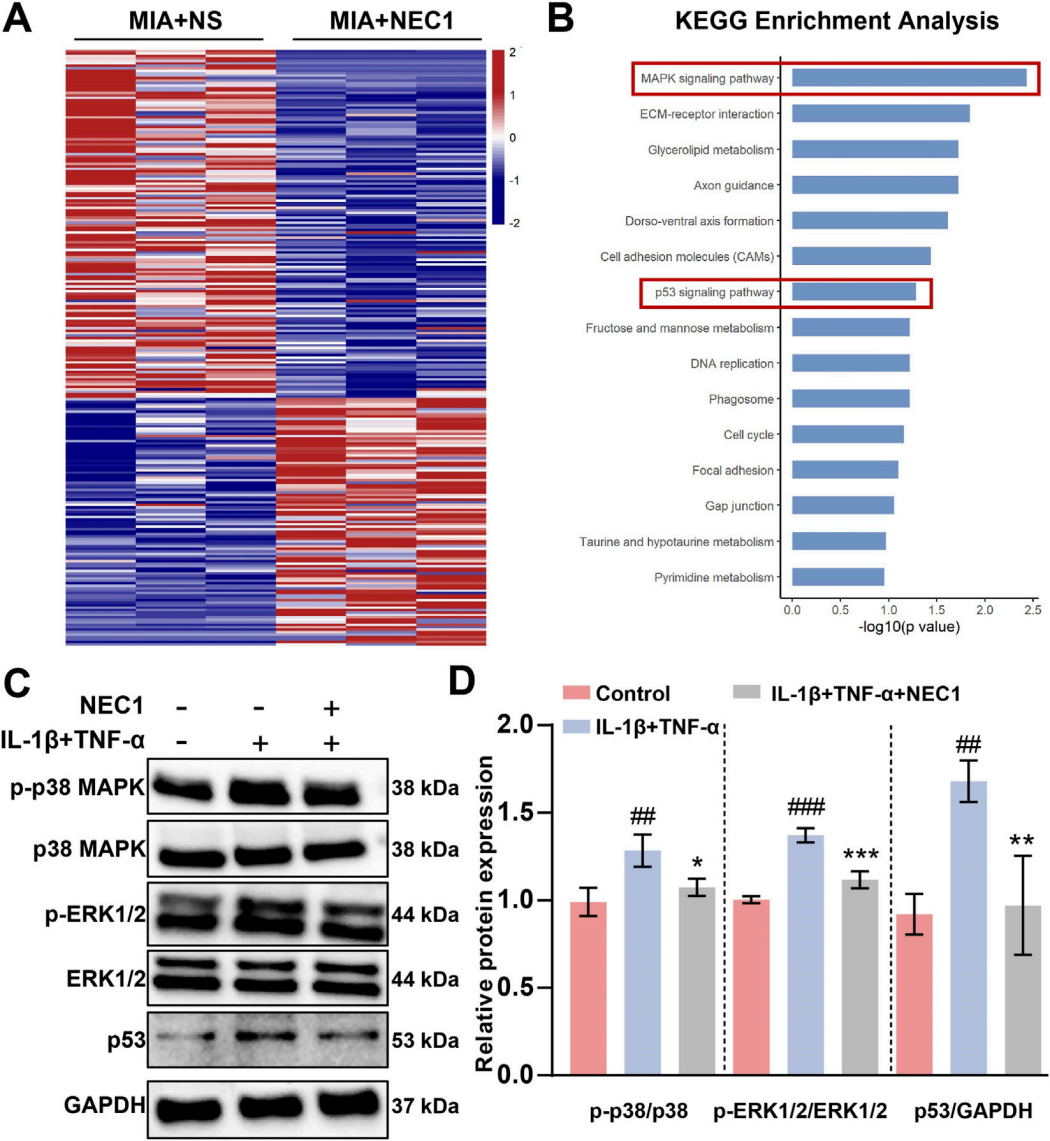
Necrostatin-1 (NEC1) inhibited p53/MAPK signaling pathway in chondrocytes. **(A)** Heatmaps of differentially expressed genes (DEGs) between untreated or treated with NEC1. **(B)** KEGG pathway enrichment analysis of DEGs. **(C,D)** p53/MAPK signaling pathway markers (p-p38 MAPK, p38 MAPK, p-ERK1/2, ERK1/2 and p53) expression and protein quantification in rPCCs (Rat primary condylar chondrocytes) were detected by Western blot. Data were analyzed by one-way ANOVA, n ≥ 3. #*p* < 0.05, ##*p* < 0.01, ###*p* < 0.001 compared to the control group; *p < 0.05, **p < 0.01, ***p < 0.001 compared to the IL1β and IL-1β and TNF-α group. ns indicates no significance.

RNA sequencing identified 354 upregulated and 337 downregulated differentially expressed genes (DEGs), with their heatmap illustrated in Figure 6A. To further elucidate the functions of these DEGs, bioinformatics analyses were performed. KEGG enrichment analysis indicated that the DEGs are primarily associated with the MAPK, ECM-receptor interaction, p53, and cell cycle signaling pathways (Figure 6B). Western blot analysis demonstrated that IL-1β and TNF-α significantly enhanced the expression of p53, phosphorylation of MAPK and ERK in rPCCs. In contrast, NEC1 treatment effectively inhibited the cytokine-induced phosphorylation of MAPK and ERK, and p53 expression (Figures 6C,D). These findings are corroborated by the mRNA sequencing results. Our data suggest that NEC1-mediated downregulation of p53, MAPK, and ERK signaling may represent a potential molecular mechanism through which it alleviates cellular senescence and progression of TMJ-OA.

## 4 Discussion

**FIGURE 7:**
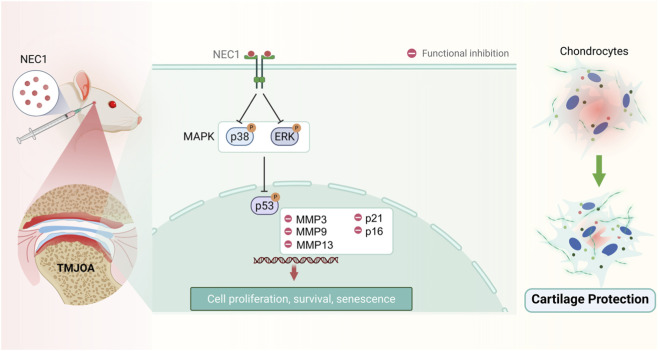
Graphical Abstract. Schematic representation of the regulatory mechanism by which Necrostatin-1 (NEC1) alleviates temporomandibular joint osteoarthritis (TMJ-OA) and inhibits chondrocyte senescence and necroptosis possibly *via* p53/MAPK downregulation.

Temporomandibular disorders (TMD) encompass a range of pathologies affecting the TMJ, with an incidence rate of 28%–40%. Among these, TMJ-OA is a notable subtype, affecting 8%–16% of the population (D. Wang et al., 2023). Major risk factors for TMJ-OA include autoimmunity, gender (particularly the influence of estrogens), mechanical overloading, inflammation, and aging (or cellular senescence), with aging being a prominent factor (Ferneini, 2021; Wu et al., 2024). In the early stages of aging, chronic, low-grade sterile inflammation has become a key feature of aging across species. Necrosis also plays a role in aging-related chronic inflammation. Approaches to block necroptosis represent a potentially new strategy to reduce “inflammaging” and retard aging (Royce, Brown-Borg and Deepa, 2019). In this study, we employed a combination of IL-1β (10 ng/mL) and TNF-α (20 ng/mL) to co-treat rPCCs. Our results demonstrated that this co-treatment scheme successfully replicated TMJ-OA pathological changes, including alterations in cell proliferation and catabolism, in a time- and concentration-dependent manner. We found that inflammation-induced condylar chondrocytes exhibited significant senescence and necroptosis, characterized by positive SA-β-gal staining and upregulation of p-MLKL (a marker of necrosis)*, Cdkn1a*, and *Cdkn2a. Cdkn1a* (p21) and *Cdkn2a* (p16) are pivotal genes involved in cell cycle regulation and the aging process. These genes play crucial roles in cellular aging by modulating the cell cycle in response to both internal and external stressors. The regulation of *Cdkn1a* and *Cdkn2a* expression is a fundamental mechanism through which cells manage stress, maintain homeostasis, and regulate the aging process. NEC1 alleviated inflammation-induced chondrocyte senescence and necrosis (Figure 7).

Currently, the management of TMJ-OA primarily includes non-surgical approaches. Non-surgical, or conservative, treatments encompass drug therapy, occlusal splint therapy, physical therapy, local injection therapy, psychological intervention, and health education. Clinically, intra-articular drug injections have proven effective in reducing pain, mitigating joint noises, and improving mandibular movement (Li and Leung, 2021). In addition to conventional injections of hyaluronic acid (HA) and hormonal treatments, emerging biological therapies—such as cell-based treatments, cell-derived exosomes, receptor antagonists or agonists, and mRNA therapeutics—hold promise for the prevention and treatment of TMJ-OA (Liu et al., 2023; Ma et al., 2020; Matheus et al., 2022; Zhang et al., 2019). However, certain treatments pose risks; for instance, hormonal drugs may exacerbate joint degeneration, and research into stem cell therapies and their derivatives faces significant ethical and practical challenges in clinical settings. Therefore, developing receptor antagonists or agonists that target key molecular pathways or cellular functions, especially those involved in cellular senescence and necrosis, may provide a viable therapeutic strategy for TMJ-OA. In our MIA-induced TMJ-OA rat model, we specifically selected female rats to better mimic the clinical characteristics of TMJ-OA (Jariyasakulroj et al., 2025; Ye et al., 2018). Numerous studies have demonstrated the efficacy of NEC1 in mitigating various *in vivo* and *in vitro* disease models, including osteoarthritis (Cao and Mu, 2021), and NEC1 has also been reported to treat TMJ-OA (He et al., 2022), which is consistent with our study that NEC1 effectively attenuates the progression of TMJ-OA in both *in vitro* and *in vivo* models.

Receptor-interacting protein kinase-1 (RIPK1) is a kinase that regulates apoptosis, necrosis, and inflammatory responses through both its kinase-dependent and kinase-independent functions, playing a crucial role in determining cell fate (Zang et al., 2019; Zhu et al., 2020). As a scaffold molecule, RIPK1 promotes the transcription of inflammatory genes by activating the mitogen-activated protein kinase (MAPK) and NF-κB pathways (Kist et al., 2021). The MAPK pathway, a key component of the serine-threonine protein kinase family, plays a critical role in the regulation of TMJ-OA by modulating inflammatory mediators (Chen et al., 2020). Furthermore, MAPK is pivotal in the regulation of MMPs and influences chondrocyte proliferation, apoptosis, and differentiation, thereby significantly contributing to the pathogenesis and progression of TMJ-OA (Fazio et al., 2024). Recent studies have suggested that the MAPK signaling pathway may negatively regulate RIPK1 (Lin et al., 2021). Activation of the MAPK signaling pathway has been shown to stimulate the secretion of MMPs in chondrocytes and osteoblasts, whereas inhibiting this pathway can alleviate TMJ-OA (Ma et al., 2020). The tumor suppressor p53 regulates DNA repair, apoptosis, cellular stress, cell cycle control, and senescence. (Hafner et al., 2019). In response to inflammatory and oxidative stress stimuli, p53 expression increases in chondrocytes, leading to chondrocyte senescence and disruptions in cartilage homeostasis (Coryell et al., 2021). Additionally, p53 can feedback regulate the MAPK signaling pathway. The p53/MAPK pathway and its associated signaling cascades, therefore, represent promising therapeutic targets in the treatment of TMJ-OA (Ma et al., 2024; Xu et al., 2020). Recent study has demonstrated that Nec1 effectively prevents chondrocyte death induced by mechanical stress. RIPK1 is a central regulator of the canonical necroptosis pathway (TNF-α–RIPK1–RIPK3–MLKL) and controls inflammation and programmed necrosis through its interaction with RIPK3. Situated downstream of TNFR1, RIPK1 directly binds to the TNFR1 death domain to mediate cell-death responses. By targeting RIPK1, Nec1 suppresses necroptosis (He et al., 2022). In this study, we found that NEC1 treatment significantly reduces chondrocyte senescence, inflammation, and necrosis during the progression of TMJ-OA, as evidenced by decreased protease secretion (MMP3, MMP9, and MMP13) and effective inhibition of the phosphorylation of MLKL, ERK, and MAPK, suggesting that this inhibition may be a key mechanism underlying the protective effects of NEC1 in TMJ-OA.

However, our current investigation into how NEC1 regulates the p53/MAPK pathways remains insufficient. On one hand, our results do not clarify which of the two signaling pathways—p53 or MAPK—plays a predominant role in the pathological progression of TMJ-OA, nor do they fully elucidate their potential crosstalk mechanisms. On the other hand, we did not perform rescue experiments (such as using p53 or MAPK agonists/inhibitors) to verify the causal relationships between these two molecular pathways. Moreover, the identification of key genes that mediate NEC1’s regulatory effects require further exploration. Therefore, the precise mechanisms by which NEC1 modulates p53, MAPK, and RIPK1 signaling in TMJ-OA need to be investigated in future studies. Relevant experiments will be conducted in our subsequent work. Importantly, future work should include the establishment of large-animal models to generate robust preclinical evidence and provide a foundation for eventual clinical translation.

## 5 Conclusion

In conclusion, NEC1 demonstrates a protective effect against TMJ-OA, as evidenced by its efficacy in the MIA-induced animal model and in mitigating inflammatory factor-induced chondrocyte damage. This protective effect appears to be mediated through the inhibition of cell senescence and necrosis and modulation of p53/MAPK signaling pathways.

## Data Availability

The RNA-seq data of this study are available in the database of NIH (https://www.ncbi.nlm.nih.gov/search/all/?term=PRJNA1375746).

## Abbreviations

BSA, bovine serum albumin; COL2A1, collagen type II alpha 1 chain; DAB, diaminobenzidine; ECM, extracellular matrix; EDTA, ethylene diamine tetraacetic acid; ERK, extracellular signal-related kinase; H&E, hematoxylin and eosin; IL-1β, interleukin-1β; JNK, c-Jun N-terminal kinase; KEGG, kyoto encyclopedia of genes and genomes; MAPK, mitogen-activated protein kinase; MMPs, metalloproteinases; MIA, monosodium iodoacetate; NEC1, necrostatin-1; NF-κB, nuclear factor-κB; NS, normal saline; PFA, paraformaldehyde; rPCCs, rat primary condylar chondrocytes; RIPA, radio immunoprecipitation assay; RIPK1:receptor interacting protein kinase-1; S&F, safranin-O and fast green; TMJ-OA, temporomandibular joint osteoarthritis; TNF-α, tumor necrosis factor-α.
